# CD48 and CXCR4 as immune-associated biomarkers in periodontitis: an integrative analysis and validation study

**DOI:** 10.3389/fimmu.2026.1861500

**Published:** 2026-07-02

**Authors:** Yue Gu, Mingyang Bu, Jingyuan Sun, Juan Liu, Lixian Yu, Xuri Yuan, Congcong Xing, Kaixuan Han, Liang Liu, XiaoDan Shen, Yakun Yang, Qing Liu

**Affiliations:** 1 Department of Pediatric Dentistry, Hebei Key Laboratory of Stomatology, Hebei Clinical Research Center for Oral Diseases, School and Hospital of Stomatology, Hebei Medical University, Shijiazhuang, China; 2 Department of Preventive Dentistry, Hebei Key Laboratory of Stomatology, Hebei Clinical Research Center for Oral Diseases, School and Hospital of Stomatology, Hebei Medical University, Shijiazhuang, China; 3 Hebei Key Laboratory of Stomatology/Hebei Technology Innovation Center of Oral Health, School and Hospital of Stomatology, Hebei Medical University, Shijiazhuang, China; 4 Department of Periodontology Dentistry, Hebei Key Laboratory of Stomatology, Hebei Clinical Research Center for Oral Diseases, School and Hospital of Stomatology, Hebei Medical University, Shijiazhuang, China; 5 Department of Pathology, The 980th Hospital of the Joint Logistics Support Force of PLA, Shijiazhuang, Hebei, China; 6 Department of Pharmacology, The Key Laboratory of Neural and Vascular Biology, Ministry of Education, The Key Laboratory of New Drug Pharmacology and Toxicology, Collaborative Innovation Center of Hebei Province for Mechanism, Diagnosis and Treatment of Neuropsychiatric Diseases, Hebei Medical University, Shijiazhuang, Hebei, China

**Keywords:** bioinformatics analysis, biomarkers, CD48, CXCR4, immune-inflammatory response, periodontitis

## Abstract

**Background:**

Periodontitis is a chronic inflammatory disease characterized by progressive destruction of periodontal supporting tissues, yet the molecular mechanisms underlying its pathogenesis remain incompletely defined. This study aimed to identify key genes associated with periodontitis through an integrative bioinformatics strategy combined with experimental validation.

**Methods:**

Two independent transcriptomic datasets (GSE16134 and GSE10334) were analyzed to identify differentially expressed genes. Weighted gene co-expression network analysis, protein–protein interaction network construction, and machine-learning approaches, including random forest and support vector machine algorithms, were employed to screen for candidate hub genes. Gene set enrichment analysis was performed to investigate potential biological functions. A ligature-induced rat periodontitis model and human periodontal tissue samples were used for validation through micro-computed tomography, histological analysis, immunohistochemistry, and quantitative real-time PCR.

**Results:**

CD48 and CXCR4 were identified as key genes consistently selected across datasets and analytical methods. Both genes were significantly upregulated in periodontitis samples and demonstrated robust diagnostic performance. Functional enrichment analysis indicated that these genes were closely associated with immune-inflammatory pathways. Experimental validation further confirmed increased expression of CD48 and CXCR4 at both mRNA and protein levels in rat and human periodontal tissues.

**Conclusion:**

CD48 and CXCR4 are potential biomarkers associated with periodontitis and may be involved in the regulation of immune-inflammatory processes, particularly those related to inflammatory cell recruitment and periodontal tissue destruction, thereby providing additional insight into disease pathogenesis and offering promising molecular candidates for future translational investigation.

## 1 Introduction

Periodontitis is a prevalent chronic inflammatory disorder affecting the supporting tissues of the teeth, including the gingiva, periodontal ligament, and alveolar bone. Progressive destruction of these structures, if left uncontrolled, ultimately leads to tooth mobility and eventual tooth loss (1, 2). Epidemiological studies have consistently demonstrated that periodontitis imposes a substantial global health burden and remains one of the leading causes of tooth loss in adults (3). Importantly, its impact extends beyond the oral cavity. A growing body of evidence has linked periodontitis to systemic conditions such as cardiovascular disease, diabetes mellitus, and rheumatoid arthritis, highlighting its broader clinical relevance (4–6). Although current therapeutic strategies primarily focus on mechanical debridement and disruption of bacterial biofilms, disease recurrence and incomplete resolution are frequently observed. This is largely attributable to the complex interplay between microbial dysbiosis and host immune responses, which remains insufficiently controlled by conventional treatments (7, 8). Consequently, elucidating the molecular mechanisms underlying periodontitis is essential for improving disease management and identifying novel therapeutic targets.

Emerging evidence indicates that dysregulation of host immune and inflammatory responses plays a pivotal role in the initiation and progression of periodontitis. In response to periodontal pathogens, the host immune system activates a complex network of signaling cascades and inflammatory mediators, which, when persistently activated, can result in excessive tissue damage (9). With the rapid development of high-throughput sequencing technologies, large-scale transcriptomic datasets have become increasingly accessible, offering new opportunities to investigate the molecular basis of complex diseases. Bioinformatics-based analytical approaches allow for systematic interrogation of gene expression profiles, facilitating the identification of disease-associated gene networks and key regulatory molecules (10, 11). In particular, integrative analytical frameworks that combine differential expression analysis, weighted gene co-expression network analysis (WGCNA), and machine-learning algorithms have been widely employed to uncover robust biomarkers and critical genes involved in disease pathogenesis (12–14). These strategies provide a powerful means to dissect the molecular landscape of periodontitis and to identify potential targets for diagnosis and therapy.

In the present study, we employed an integrative bioinformatics approach to identify genes closely associated with periodontitis. By combining analyses of multiple independent transcriptomic datasets with experimental validation in both animal models and human tissues, we aimed to screen for reliable molecular candidates and to further characterize their potential roles in periodontal inflammation. This work is expected to provide additional insights into the molecular mechanisms of periodontitis and to offer potential directions for future diagnostic and therapeutic development.

## 2 Methods and materials

### 2.1 Data acquisition

Two publicly available gene expression datasets related to periodontitis (GSE16134 and GSE10334) were retrieved from the Gene Expression Omnibus (GEO) database (https://www.ncbi.nlm.nih.gov/geo/). Both datasets were generated using the GPL570 platform and include gingival tissue samples from patients with periodontitis and healthy controls. The GSE16134 dataset comprises 310 samples (69 healthy and 241 periodontitis), while GSE10334 contains 247 samples (64 healthy and 183 periodontitis). Raw expression data and corresponding platform annotation files were downloaded for subsequent analyses.

### 2.2 Identification of differentially expressed genes

Differential expression analysis between periodontitis and healthy gingival tissues was performed using the limma package (version 3.54.0) in R software (version 4.2.0). Raw expression data were normalized and log2-transformed prior to analysis. Probe annotation was conducted based on the platform annotation files, and probes without corresponding gene symbols were excluded. For genes mapped by multiple probes, the average expression value was calculated (15).

Differentially expressed genes (DEGs) were identified using a linear model combined with empirical Bayes moderation. P values were adjusted using the Benjamini–Hochberg method to control the false discovery rate (FDR). Genes with |log2 fold change| > 1 and adjusted P < 0.05 were considered statistically significant. Volcano plots were generated using the ggplot2 package to visualize DEG distribution (16).

### 2.3 Weighted gene co-expression network analysis

Weighted gene co-expression network analysis (WGCNA) was performed using the WGCNA package (version 1.72-1) to identify gene modules associated with periodontitis. Samples with excessive missing values were excluded. An appropriate soft-thresholding power was selected based on the criterion of approximate scale-free topology. The adjacency matrix was subsequently transformed into a topological overlap matrix (TOM) to evaluate gene connectivity.

Gene modules were identified using hierarchical clustering combined with the dynamic tree-cutting algorithm (17). Module–trait relationships were calculated to assess correlations between gene modules and disease status. The module showing the strongest association with periodontitis was defined as the key module. Gene significance (GS) and module membership (MM) were further calculated to identify genes highly correlated with both the phenotype and the module (18).

### 2.4 Protein–protein interaction network construction

To investigate potential interactions among candidate genes, a protein–protein interaction (PPI) network was constructed using the STRING database (version 11.5; https://string-db.org/) (19). Overlapping genes identified from DEG and WGCNA analyses were submitted with the species restricted to Homo sapiens. An interaction score > 0.4 was used as the cutoff, and disconnected nodes were excluded.

The resulting network was imported into Cytoscape software (version 3.9.1) for visualization and topological analysis. The CytoHubba plugin was used to evaluate node importance within the network (20). Genes were ranked using Degree and Betweenness centrality algorithms, and those consistently ranked highly by both methods were defined as hub genes.

### 2.5 Machine learning-based identification of key genes

To further refine candidate biomarkers, machine-learning algorithms including random forest (RF) and support vector machine (SVM) were applied to the hub genes. RF analysis was conducted using the randomForest package (version 4.7-1.1), and SVM analysis was performed using the e1071 package (version 1.7-13).

In the RF model, feature importance was assessed based on the mean decrease in accuracy, and the number of trees was set to 500 (21). In the SVM model, genes with high discriminative ability between periodontitis and control samples were selected (22). Candidate genes identified by both algorithms in the two datasets were intersected to obtain robust key genes.

### 2.6 Receiver operating characteristic curve analysis

Receiver operating characteristic (ROC) curve analysis was performed to evaluate the diagnostic performance of key genes using the pROC package (version 1.18.0) in R (23). The area under the curve (AUC) was calculated as an indicator of discriminative ability. In general, AUC values of 0.7–0.8 indicate acceptable performance, 0.8–0.9 indicate good performance, and values greater than 0.9 indicate excellent diagnostic accuracy.

### 2.7 Gene set enrichment analysis

Gene Set Enrichment Analysis (GSEA) was conducted using the clusterProfiler package (version 4.6.0) to explore potential biological functions of the identified key genes. Samples were divided into high- and low-expression groups according to the median expression level of each gene. The Kyoto Encyclopedia of Genes and Genomes (KEGG) database was used as the reference gene set. Pathways with adjusted P < 0.05 were considered significantly enriched (24).

### 2.8 Establishment of the rat periodontitis model

Male Sprague–Dawley rats (8 weeks old) were housed under specific pathogen-free conditions with free access to food and water. Animals were randomly assigned to a control group (n = 6) and a periodontitis group (n = 6). All experimental procedures were approved by the Animal Ethics Committee of the Hebei Medical University (NO. 2026375) and conducted in accordance with relevant guidelines.

Experimental periodontitis was induced using a ligature model. Briefly, under anesthesia, a sterile silk ligature was placed around the maxillary second molar to promote plaque accumulation and induce periodontal inflammation. The ligatures were maintained for 20 days to allow disease development (25). Control animals received no ligature. After the experimental period, rats were sacrificed and maxillary tissues were harvested for subsequent analyses.

### 2.9 Micro-computed tomography analysis

Alveolar bone morphology was evaluated using micro-computed tomography (micro-CT). Maxillary specimens were fixed in 4% paraformaldehyde and scanned at 70 kV and 114 μA with a voxel resolution of 9 μm. Three-dimensional reconstruction and quantitative analyses were performed using the corresponding software.

The following parameters were assessed: bone mineral density (BMD), bone volume (BV), bone volume fraction (BV/TV), trabecular thickness (Tb.Th), trabecular number (Tb.N), and trabecular separation (Tb.Sp). Alveolar bone loss was quantified by measuring the distance from the cementoenamel junction (CEJ) to the alveolar bone crest (ABC).

### 2.10 Human periodontal tissue samples

Human periodontal tissues were obtained from patients treated at the Stomatological Hospital of Hebei Medical University. Periodontitis samples were collected from patients diagnosed with periodontitis, whereas healthy gingival tissues obtained during orthodontic extraction served as controls. A total of 40 samples (20 periodontitis and 20 healthy) were included.

All participants provided written informed consent. The study was approved by the Ethics Committee of the Stomatological Hospital of Hebei Medical University (NO. 2024023) and conducted in accordance with the Declaration of Helsinki.

### 2.11 Histological analysis (H&E staining)

Rat and human periodontal tissues were fixed in 4% paraformaldehyde. Rat maxillae were decalcified in 10% EDTA solution prior to processing. Tissues were dehydrated, paraffin-embedded, and sectioned at 4–5 μm thickness. Sections were stained with hematoxylin and eosin (H&E) following standard protocols. Histopathological changes were observed under a light microscope.

### 2.12 Immunohistochemistry

Immunohistochemistry was performed to detect CD48 and CXCR4 protein expression. Sections were deparaffinized, rehydrated, and subjected to antigen retrieval using citrate buffer. Endogenous peroxidase activity was blocked with 3% hydrogen peroxide.

Sections were incubated with primary antibodies against CD48 and CXCR4 (Abcam, UK) overnight at 4 °C, followed by incubation with appropriate secondary antibodies. Immunoreactivity was visualized using 3,3′-diaminobenzidine (DAB), and nuclei were counterstained with hematoxylin. Staining intensity was quantified as mean optical density (MOD) using ImageJ software.

### 2.13 Quantitative real-time PCR

Total RNA was extracted using TRIzol reagent (Invitrogen, USA) according to the manufacturer’s instructions. RNA concentration and purity were measured spectrophotometrically. One microgram of RNA was reverse-transcribed into cDNA.

Quantitative real-time PCR (qPCR) was performed using SYBR Green Master Mix. Relative gene expression levels were normalized to GAPDH (Gapdh for rat) and calculated using the 2^-ΔΔCt method.

Primer sequences were as follows:

Rat (5′–3′):

Cd48: F, TGTGCTGCTGCTACTTTGGA; R, AGGTGACGATGTTGGAGTGA.

Cxcr4: F, AGGAAACTGCTGGCTGAAAAGG; R, GGAATTGAAACACCACCATCCA.

Gapdh: F, AGGTCGGTGTGAACGGATTTG; R, TGTAGACCATGTAGTTGAGGTCA.

Human (5′–3′):

CD48: F, GCTTGAAACCACCCTTATGCCAC; R, CGTGACCACTAGCCAACTTGCA.

CXCR4: F, CTCCTCTTTGTCATCACGCTTCC; R, GGATGAGGACACTGCTGTAGAG.

GAPDH: F, GTCTCCTCTGACTTCAACAGCG; R, ACCACCCTGTTGCTGTAGCCAA.

### 2.14 Statistical analysis

Statistical analyses were performed using R software (version 4.2.0) and GraphPad Prism (version 9.0). Data are presented as mean ± standard deviation (SD). Differences between two groups were analyzed using Student’s t-test. A two-tailed P value < 0.05 was considered statistically significant.

## 3 Results

### 3.1 Identification of periodontitis-associated gene modules by differential expression analysis and WGCNA

**Figure 1:**
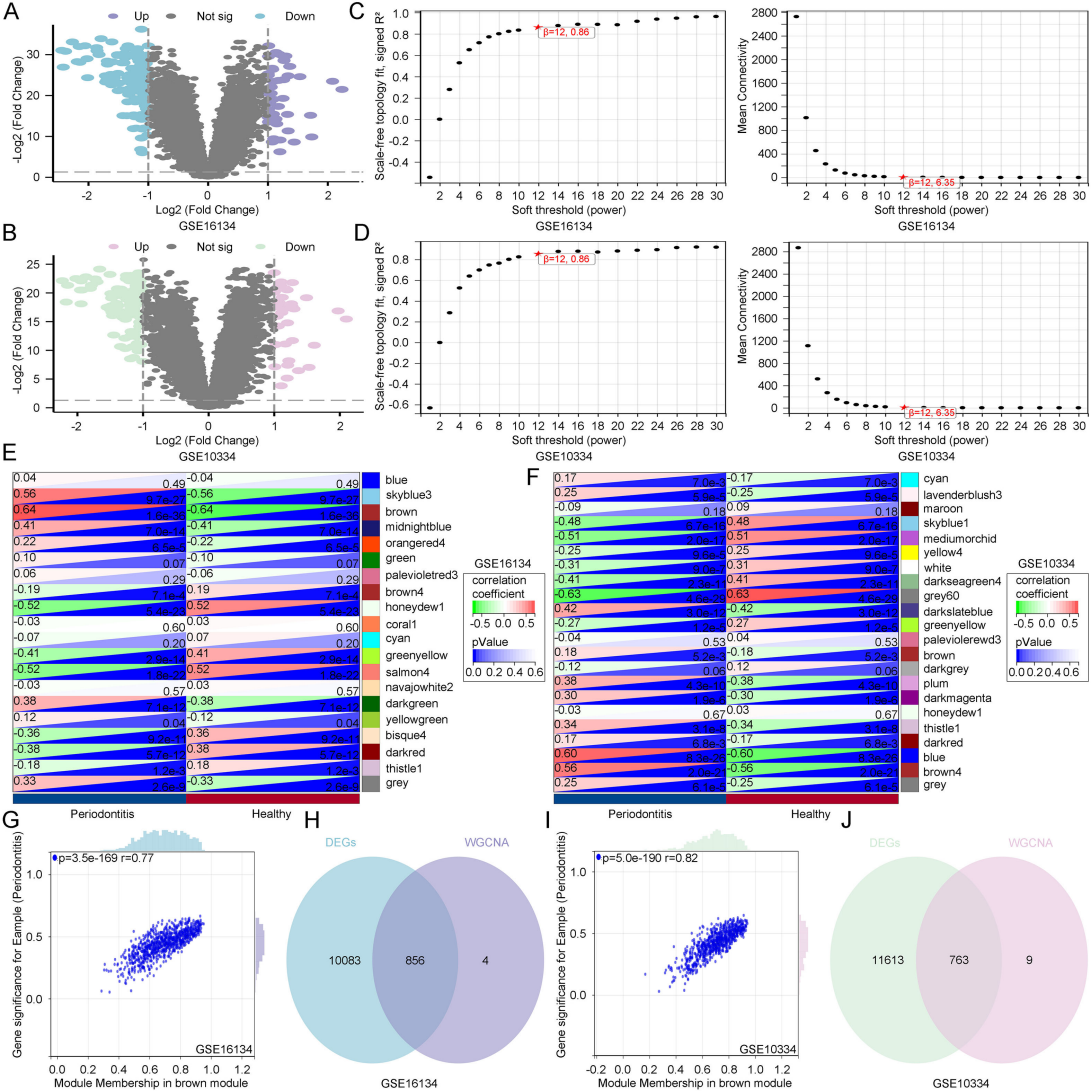
Identification of periodontitis-related gene modules by DEGs and WGCNA. **(A, B)** Volcano plots of DEGs in GSE16134 and GSE10334. **(C, D)** Soft-threshold determination for scale-free network construction. **(E, F)** Module–trait correlations showing the relevant module. **(G, I)** Correlation of gene significance with module membership in the brown module. **(H, J)** Venn diagrams of overlapping genes between DEGs and the brown module.

To identify genes associated with periodontitis, differential expression analysis was performed using two independent transcriptomic datasets, GSE16134 and GSE10334. Volcano plots demonstrated marked transcriptional differences between periodontitis and healthy gingival tissues in both datasets, indicating widespread gene expression alterations during disease progression (**Figures 1A, B**).

To further explore co-expression patterns related to disease status, WGCNA was conducted in both datasets (**Figures 1C, D**). Module–trait relationship analysis identified 14 modules significantly associated with the periodontitis phenotype in GSE16134 (**Figure 1E**) and 19 modules in GSE10334 (**Figure 1F**). Consistently, correlation analysis between gene significance (GS) and module membership (MM) showed that the brown module exhibited the strongest GS–MM association in both datasets (r = 0.77 in GSE16134; r = 0.82 in GSE10334; **Figures 1G, I**). These findings suggest that genes within the brown module are highly relevant to the periodontitis phenotype.

Based on these observations, the brown module was selected for further analysis. To enhance the reliability of candidate gene selection, DEGs were intersected with genes within the brown module in each dataset. This approach yielded 856 overlapping genes in GSE16134 (**Figure 1H**) and 763 genes in GSE10334 (**Figure 1J**), which were subsequently used for downstream network and machine-learning analyses.

### 3.2 Identification of periodontitis-related hub genes by PPI network topology analysis

**Figure 2:**
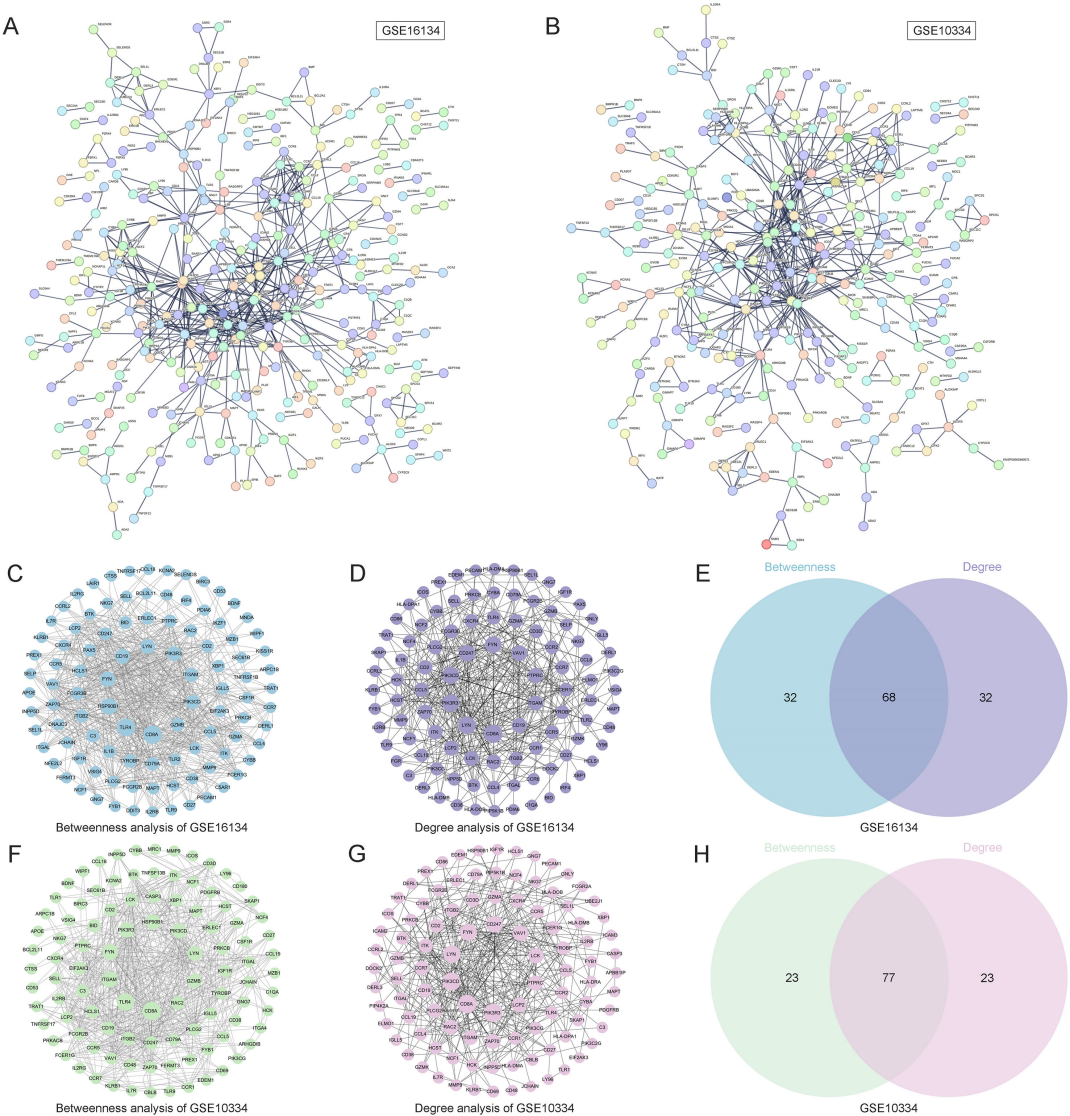
Identification of periodontitis-related hub genes by PPI network topology analysis. **(A, B)** PPI networks constructed from overlapping genes in GSE16134 and GSE10334. **(C, D)** Hub genes identified by Betweenness and Degree analyses in GSE16134. **(E)** Overlapping hub genes identified by the two algorithms in GSE16134. **(F, G)** Hub genes identified by Betweenness and Degree analyses in GSE10334. **(H)** Overlapping hub genes identified by the two algorithms in GSE10334.

To further refine candidate genes, protein–protein interaction (PPI) networks were constructed based on the overlapping genes identified in GSE16134 and GSE10334 (**Figures 2A, B**). Network topology analysis was performed using Degree and Betweenness centrality algorithms to identify key nodes within each network.

In GSE16134, both algorithms identified a set of highly connected genes (**Figures 2C, D**), and their intersection yielded 68 common hub genes (**Figure 2E**). Similarly, in GSE10334, 77 hub genes were identified based on the overlap of the two topological methods (**Figures 2F–H**). The convergence of results across different algorithms supports the robustness of hub gene selection.

These hub genes were retained for subsequent machine-learning analysis to further identify key genes with potential diagnostic relevance.

### 3.3 Identification and evaluation of key genes in periodontitis by machine-learning algorithms

**Figure 3:**
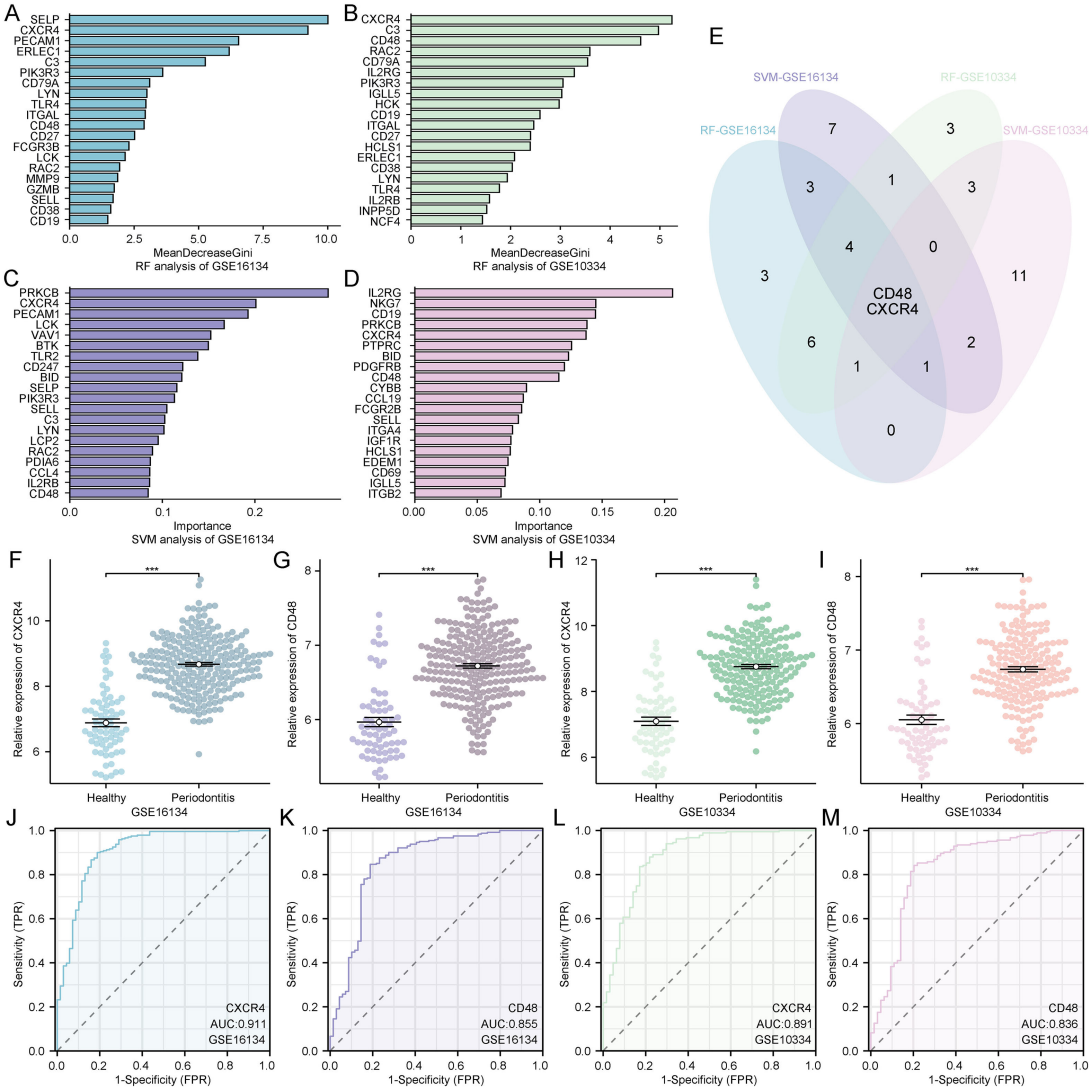
Identification of key genes in periodontitis by machine-learning algorithms. **(A, B)** Important genes identified by random forest analysis in GSE16134 and GSE10334. **(C, D)** Important genes identified by SVM analysis in GSE16134 and GSE10334. **(E)** Overlapping genes identified by RF and SVM in both datasets. **(F–I)** Expression levels of CXCR4 and CD48 in GSE16134 and GSE10334. **(J–M)** ROC curves evaluating the diagnostic performance of CXCR4 and CD48 in the two datasets. ***p < 0.001.

To further identify genes with diagnostic potential, random forest (RF) and support vector machine (SVM) algorithms were applied to the hub genes in both datasets. Each method identified a subset of genes with relatively high importance scores (**Figures 3A–D**).

To improve the robustness of feature selection, candidate genes identified by RF and SVM across both datasets were intersected. Only two genes, CD48 and CXCR4, were consistently retained (**Figure 3E**), indicating that these genes may represent stable biomarkers associated with periodontitis.

The expression levels of CD48 and CXCR4 were subsequently examined. Both genes were significantly upregulated in periodontitis samples compared with healthy controls in GSE16134 and GSE10334 (**Figures 3F–I**). ROC curve analysis further demonstrated their diagnostic performance. CXCR4 showed AUC values of 0.911 and 0.891, whereas CD48 showed AUC values of 0.855 and 0.836 in GSE16134 and GSE10334, respectively (**Figures 3J–M**). These results indicate that both genes possess good discriminative ability for distinguishing periodontitis from healthy tissues.

### 3.4 GSEA reveals close associations of CD48 and CXCR4 with immune-inflammatory pathways

**Figure 4:**
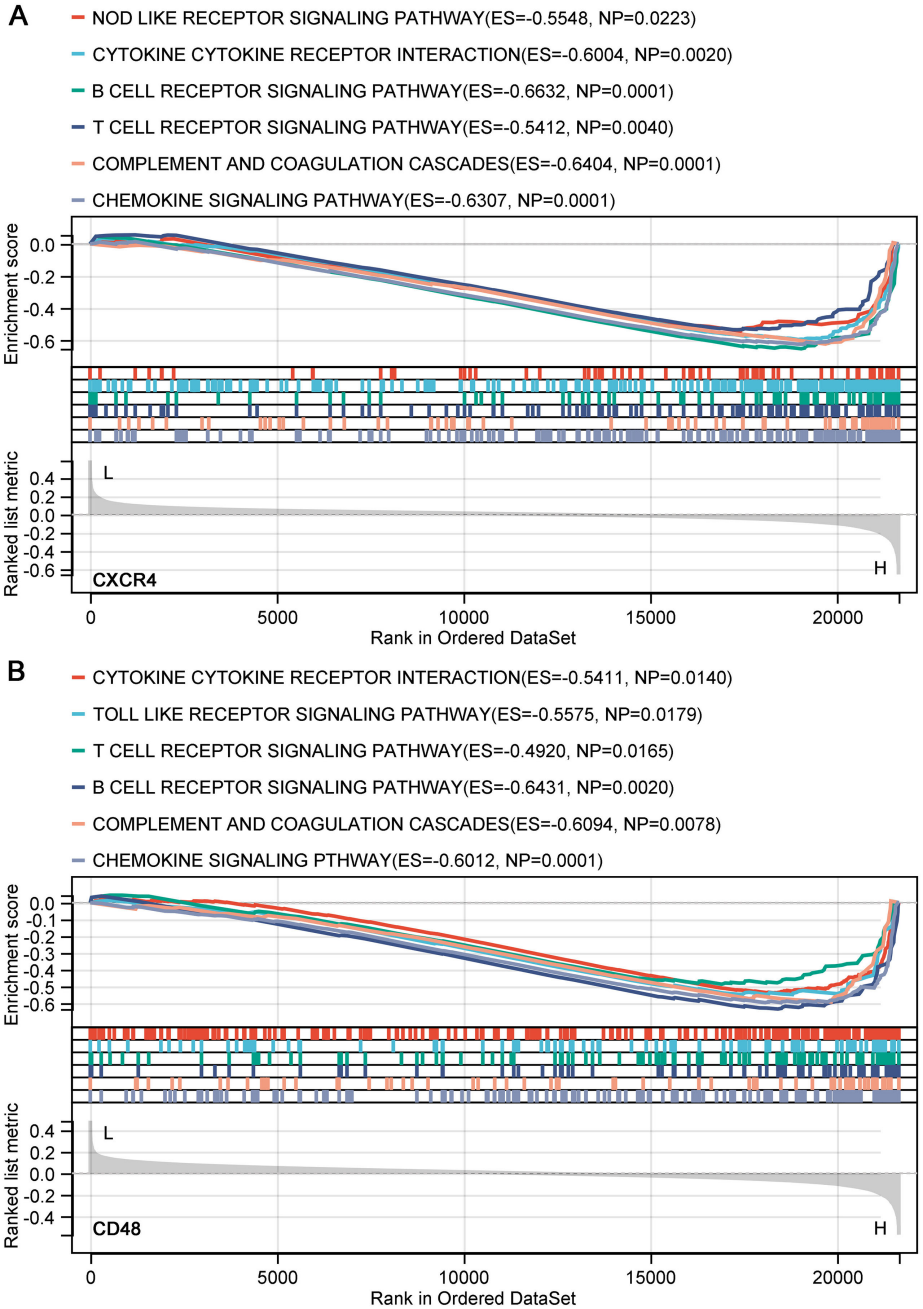
GSEA of CXCR4 and CD48 in periodontitis. **(A)** Enriched signaling pathways associated with CXCR4. **(B)** Enriched signaling pathways associated with CD48.

To investigate the biological relevance of CD48 and CXCR4, GSEA was performed based on their expression levels. For both genes, the high-expression groups were significantly enriched in multiple pathways associated with immune and inflammatory responses (**Figures 4A, B**).

Notably, enriched pathways included cytokine–cytokine receptor interaction, chemokine signaling, B cell receptor signaling, T cell receptor signaling, complement and coagulation cascades, as well as NOD-like receptor and Toll-like receptor signaling pathways. These findings indicate that elevated expression of CD48 and CXCR4 is closely associated with immune-inflammatory processes in periodontitis.

### 3.5 Experimental validation of CD48 and CXCR4 upregulation in rat and human periodontal tissues

**Figure 5:**
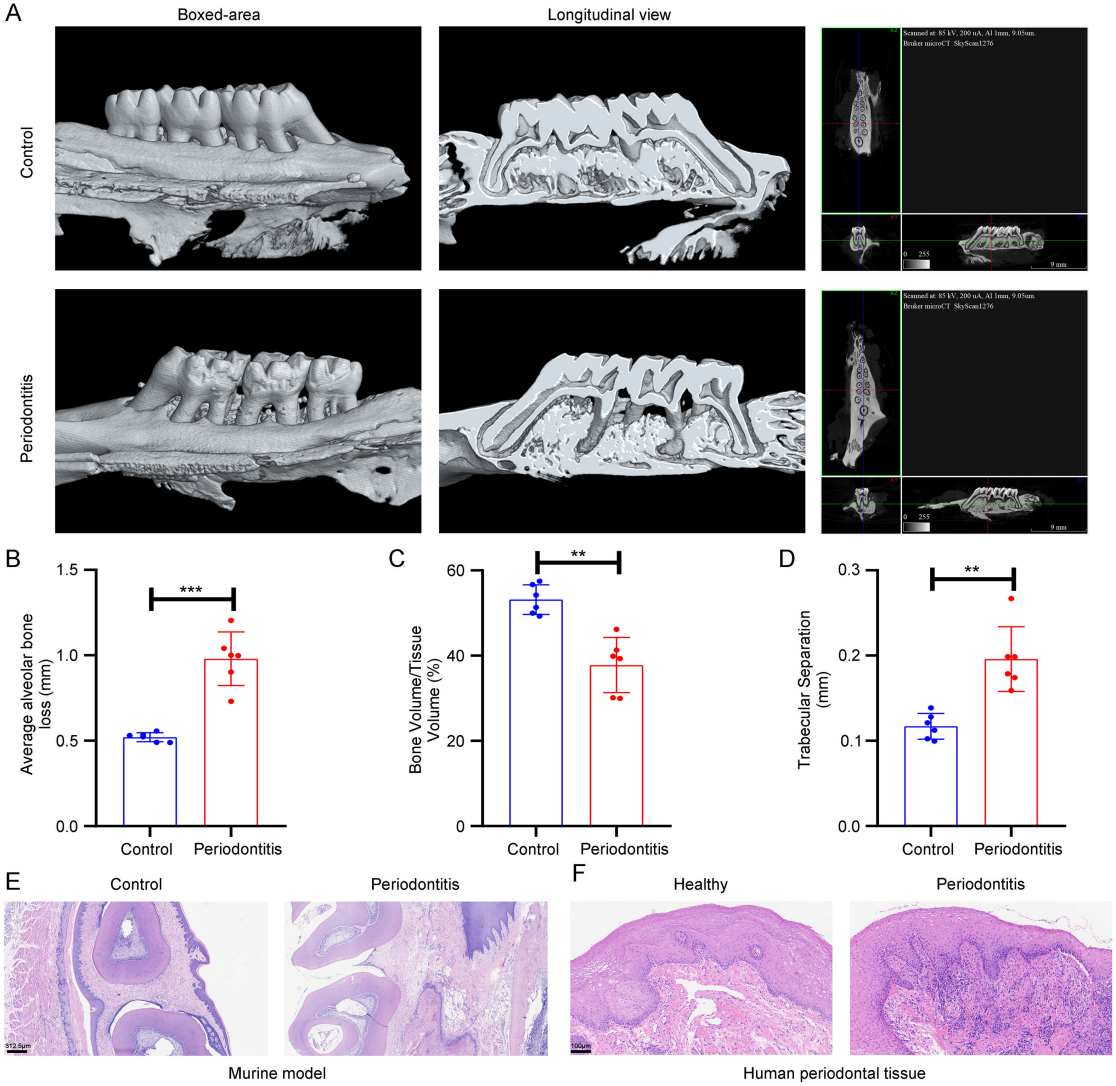
Validation of the rat periodontitis model and histopathological changes in rat and human periodontal tissues. **(A)** Representative micro-CT images of the control and periodontitis groups. **(B–D)** Quantitative analysis of alveolar bone loss, bone volume fraction, and trabecular separation. **(E)** Representative H&E staining of rat periodontal tissues. **(F)** Representative H&E staining of human periodontal tissues. **p < 0.01, ***p < 0.001 versus the control group.

To validate the bioinformatics findings, a ligature-induced periodontitis model was established in rats, and human periodontal tissue samples were collected. Micro-CT analysis showed that rats in the periodontitis group exhibited pronounced alveolar bone resorption, including increased alveolar bone loss, reduced bone volume fraction, and increased trabecular separation compared with controls (**Figures 5A–D**).

Consistently, H&E staining revealed more severe inflammatory infiltration and tissue destruction in both rat and human periodontitis tissues relative to their respective control groups (**Figures 5E, F**), confirming successful model establishment and the presence of characteristic pathological features.

**Figure 6:**
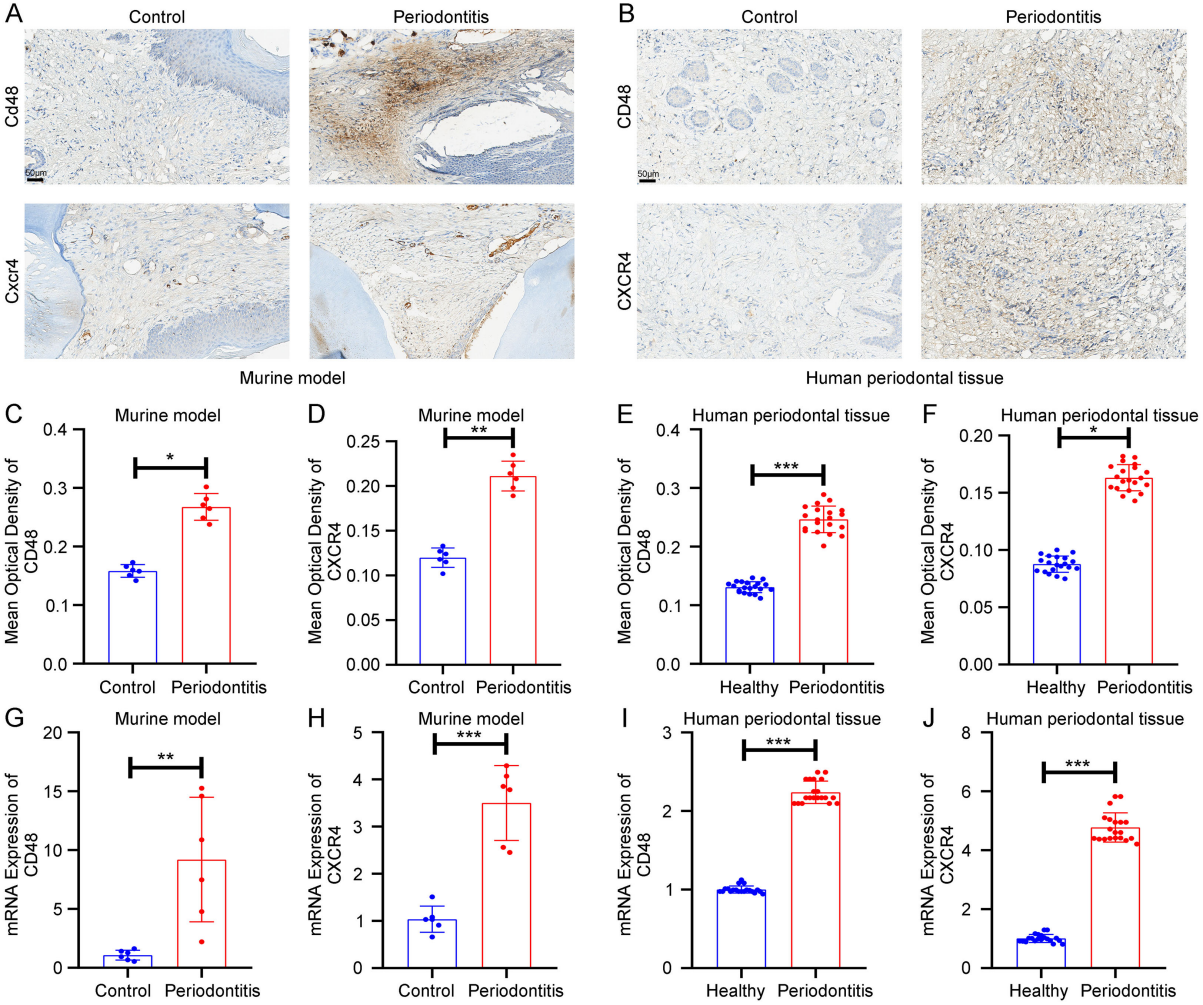
Experimental validation of CD48 and CXCR4 expression in rat and human periodontal tissues. **(A, B)** Representative immunohistochemical staining of CD48 and CXCR4 in rat and human periodontitis tissues. **(C–F)** Quantification of mean optical density for CD48 and CXCR4. **(G–J)** qPCR analysis of CD48 and CXCR4 mRNA expression in rat and human periodontitis tissues. *p < 0.05, **p < 0.01, ***p < 0.001 versus the corresponding control group.

Immunohistochemical analysis demonstrated that CD48 and CXCR4 protein expression levels were markedly elevated in periodontitis tissues compared with controls (**Figures 6A, B**). Quantitative analysis of mean optical density further confirmed significant upregulation of both proteins in rat and human samples (**Figures 6C–F**). In addition, qPCR results showed that CD48 and CXCR4 mRNA levels were significantly increased in periodontitis tissues (**Figures 6G–J**).

These findings collectively demonstrate that CD48 and CXCR4 are consistently upregulated in periodontitis across both experimental models and clinical samples.

## 4 Discussion

Periodontitis is a chronic inflammatory disease characterized by progressive destruction of the periodontal ligament and alveolar bone, ultimately resulting in tooth loss if not effectively controlled (26). Although substantial progress has been made in periodontal therapy, current treatment still relies largely on mechanical debridement and biofilm control, which are often insufficient to completely prevent disease progression or recurrence. This limitation is largely attributable to the complex interplay between microbial dysbiosis and host immune responses (27). Against this background, identification of molecular regulators involved in periodontal inflammation remains of considerable importance. In the present study, integrative bioinformatics analyses of two independent transcriptomic datasets, together with experimental validation in rat and human periodontal tissues analyze, identified CD48 and CXCR4 as potential key genes associated with periodontitis. Both genes were consistently upregulated in diseased tissues, supporting their potential relevance to the inflammatory microenvironment of periodontitis.

CD48 is a glycosylphosphatidylinositol-anchored membrane protein belonging to the signaling lymphocyte activation molecule (SLAM) family (28). It is broadly expressed on immune cells and has been implicated in immune-cell activation, adhesion, and inflammatory regulation (29). Previous studies have shown that CD48 participates in immune signaling and contributes to inflammatory processes in a range of immune-mediated disorders, including asthma and autoimmune diseases (30, 31). In the context of periodontitis, where sustained immune activation and inflammatory-cell infiltration are central pathological features, increased CD48 expression may reflect enhanced local immune activity within periodontal lesions. It is also plausible that CD48 contributes to the amplification of inflammatory signaling, thereby promoting persistence of periodontal inflammation. From this perspective, the elevated CD48 expression observed in our study may not merely represent a secondary change, but may also suggest an active role in shaping the local immune response.

The second key gene identified in this study, CXCR4, is a chemokine receptor involved in leukocyte trafficking, inflammatory-cell recruitment, and tissue remodeling (32). The CXCL12/CXCR4 axis has been widely implicated in inflammatory and immune-related diseases and is known to regulate immune-cell migration and inflammatory responses (33, 34). Previous evidence has also suggested that CXCR4 signaling may participate in periodontal inflammation by facilitating immune-cell recruitment into periodontal tissues (35). In this regard, the increased CXCR4 expression detected in both the public datasets and the experimental samples is biologically plausible. Given the importance of inflammatory-cell accumulation in periodontal tissue destruction, upregulation of CXCR4 may contribute to maintenance of the chronic inflammatory state that characterizes periodontitis. In addition, because chemokine signaling is closely linked to tissue remodeling, CXCR4 may also be involved in the structural alterations associated with periodontal breakdown.

The enrichment results further supported the involvement of CD48 and CXCR4 in immune-inflammatory regulation. Genes associated with high expression of these markers were enriched in pathways related to cytokine–cytokine receptor interaction, chemokine signaling, and lymphocyte activation. These pathways are closely connected to immune dysregulation and inflammatory injury in periodontal disease (36, 37). Recent transcriptomic studies have likewise emphasized that immune-related signaling networks constitute a major component of the molecular landscape of periodontitis and may offer promising candidates for biomarker discovery and therapeutic intervention signaling (38, 39). Accordingly, the present findings suggest that CD48 and CXCR4 are unlikely to act in isolation; rather, they may participate in broader inflammatory networks that contribute to periodontal tissue destruction. This interpretation is also consistent with the view that periodontitis is driven not only by microbial challenge, but also by dysregulated host responses. In this context, CD48 and CXCR4 may represent molecular links between immune activation and structural damage in periodontal tissues.

Several limitations of this study should be acknowledged. First, although two independent public datasets were included to enhance the robustness of bioinformatics screening, the findings may still be influenced by sample heterogeneity and differences in dataset composition. Second, the number of animal and human samples used for validation was relatively limited, which may restrict the generalizability of the experimental observations. Third, while the present study established a close association between CD48/CXCR4 expression and periodontitis, the precise mechanisms through which these genes contribute to periodontal inflammation remain unresolved. Further functional studies will therefore be necessary to determine whether these genes act as causal regulators of disease progression or primarily reflect the inflammatory state of periodontal tissues.

Taken together, the present study supports CD48 and CXCR4 as potential biomarkers associated with periodontitis. Their consistent upregulation in public datasets, rat periodontal tissues, and human clinical samples, together with their close association with immune-inflammatory pathways, suggests that they may be involved in key molecular events underlying periodontal inflammation. These findings expand the current understanding of periodontitis pathogenesis and may provide a basis for future studies aimed at biomarker development and mechanism-based therapeutic intervention. More importantly, they support the possibility that targeting immune-associated signaling molecules may help limit the destructive inflammatory responses that drive periodontal breakdown signaling.

## 5 Conclusions

This study combined integrative transcriptomic analyses of independent public datasets with experimental validation in rat and human periodontal tissues to identify CD48 and CXCR4 as genes associated with periodontitis. Both genes were consistently upregulated at the mRNA and protein levels and showed close associations with immune-inflammatory signaling pathways. These findings suggest that CD48 and CXCR4 may participate in key processes underlying periodontal inflammation and tissue destruction, and highlight their promise as candidate biomarkers and targets for further investigation. Our results also underscore the potential translational relevance of immune-associated biomarkers in improving the molecular understanding and future management of periodontitis.

## Data Availability

The original contributions presented in the study are included in the article/supplementary material. Further inquiries can be directed to the corresponding author/s.
